# Seeing Circles and Drawing Ellipses: When Sound Biases Reproduction of Visual Motion

**DOI:** 10.1371/journal.pone.0154475

**Published:** 2016-04-27

**Authors:** Etienne Thoret, Mitsuko Aramaki, Lionel Bringoux, Sølvi Ystad, Richard Kronland-Martinet

**Affiliations:** 1 Laboratoire de Mécanique et d’Acoustique, CNRS, UPR 7051, Aix Marseille Université, Centrale Marseille, Marseille, France; 2 Aix-Marseille Université, CNRS, ISM, UMR 7287, Marseille, France; State University of New York Downstate Medical Center, UNITED STATES

## Abstract

The perception and production of biological movements is characterized by the 1/3 power law, a relation linking the curvature and the velocity of an intended action. In particular, motions are perceived and reproduced distorted when their kinematics deviate from this biological law. Whereas most studies dealing with this perceptual-motor relation focused on visual or kinaesthetic modalities in a unimodal context, in this paper we show that auditory dynamics strikingly biases visuomotor processes. Biologically consistent or inconsistent circular visual motions were used in combination with circular or elliptical auditory motions. Auditory motions were synthesized friction sounds mimicking those produced by the friction of the pen on a paper when someone is drawing. Sounds were presented diotically and the auditory motion velocity was evoked through the friction sound timbre variations without any spatial cues. Remarkably, when subjects were asked to reproduce circular visual motion while listening to sounds that evoked elliptical kinematics without seeing their hand, they drew elliptical shapes. Moreover, distortion induced by inconsistent elliptical kinematics in both visual and auditory modalities added up linearly. These results bring to light the substantial role of auditory dynamics in the visuo-motor coupling in a multisensory context.

## Introduction

It is now well established that biological motion is characterized by specific kinematic properties, for instance, by the 1/3 power law which postulates that the tangential velocity of the motion *v*_*t*_ is constrained by the local curvature C of its geometrical trajectory: *v*_*t*_ *= KC*^*-1/3*^ with K a constant [1]. Regarding the visual modality, it has been shown that the human ability to track a visual motion with the non-hidden hand is facilitated when the motion complies with the 1/3 power law [2]. By contrast, the perceived geometry of a circular visual motion can be distorted if the motion does not comply with these biological rules [3]. More recently, we showed that the visuo-motor coupling of circular motions displayed with incongruent elliptical kinematics were distorted by subjects who do not see their hand [4]. Regarding the kinaesthetic modality, Viviani, Baud-Bovy, and Redolfi [5] have also shown that the perception of the movement geometry is constrained by the covariations between the movement kinematics and its curvature. Indeed, a circular hand movement trailed by a mechanical arm that does not comply with the 1/3-power law distorts its perceived geometry into an elliptical one. Taken together, these results confirmed the existing relation between curvature and dynamics in the emergence of the perceived and/or reproduced motion geometry.

In the field of multisensory research, many studies have demonstrated the ability of the auditory modality to modify the visual perception of motion [6,7] (see [8] for a review) and even drive our motor behavior [9] (see [10] for a review). For instance, Brooks et al. [11] showed that the visual perception of a specific kind of biological movement, represented by a point-like walker, is affected by spatial auditory motion. In line with this observation, Arrighi et al. [12] demonstrated that sounds can even enhance the visual perception of point-like walkers when displayed with synchronous auditory motions. These studies give credit to a multisensory processing framework of biological motions.

The present study investigated the influence of movement dynamics on the reproduced geometry of biological motion by combining auditory and visual stimuli evoking circular or elliptical motions in consistent or inconsistent audiovisual situations. Whereas spatial (i.e. geometrics-related) cues are intrinsically conveyed by visual (and kinaesthetic) sensory inputs, we hereby investigated purely dynamic (i.e., kinematics-related) cues without spatial information by using monophonic sounds [13]. Indeed, recent experiments revealed that time-varying, monophonic friction sounds produced by someone drawing evoke the movement kinematics of the drawer’s pencil and enable, to a certain extent, the recognition of the drawn shape [14]. In our experiment, subjects were asked to synchronize drawing movements on a graphic tablet with visual motions displayed on a screen without seeing their hand. It was hypothesized that sounds evoking incongruent kinematics with respect to the visual motion affect the geometry of the reproduced shape resulting from the coupling between a visual biological motion and a drawing movement. This would highlight the role of movement dynamics in the emergence of the geometry independently from spatial cues.

By investigating the effect of congruent and incongruent combinations of auditory and visual kinematics on visuo-motor coupling of biological motion, we aimed at considering crossmodal influences of these two modalities. As previously assumed, if movement dynamics alone influence the geometry of the motion induced by such a visuo-motor coupling, it would allow for the investigation of the combined role of the auditory and visual channels in a multisensory context.

## Materials & Methods

### Participants

Seventeen right-handed subjects (2 women; M = 28.5 years, SD = 8 years) participated in the experiment. All subjects provided their consent prior to the study and were naive as to the specific purpose of the experiment. At the time the experiment was designed and conducted (winter 2012–2013), no ethics approval was required from the Aix-Marseille University for behavioral studies such as those reported in this manuscript. The local Ethics board at the Aix-Marseille University subsequently approved highly similar experiments conducted in the same Institute two years later the experiment reported in this manuscript was conducted. Neither of the experiments involved deception or stressful procedures. Participants were informed that they were free to leave the experiment at any time, and that their data would have been treated anonymously. The research reported in this manuscript was carried out according to the principles expressed in the 1964 Declaration of Helsinki. Participants in the experiment were recruited on a voluntary basis from the students and staff of the Groupement des Laboratoires de Marseille. All the data were anonymized before analysis.

### Stimuli

Audiovisual stimuli evoking motions were generated from specific kinematic rules applied to both visual and auditory stimuli leading to consistent or inconsistent situations. According to the kinematic behavior of planar motion, the dynamic behavior was modeled by a system of two harmonic oscillators of period T that differ by their relative phase, noted Φ, allowing for the simulation of specific kinematic properties ranging from circular to elliptical motion [13]:

$$\left\{ \begin{array}{l} x(s(t))=Amp\cdot \text{cos}\left(\frac{2\pi }{T}s(t)\right)\\ y(s(t))=Amp\cdot \text{cos}\left(\frac{2\pi }{T}s(t)+\Phi \right) \end{array}\right.$$

where *Amp* is the amplitude of the motion, x and y the coordinates of the motion in the (x(t), y(t)) plane, s(t) the curvilinear abscissa along the trajectory, and t the time. Interestingly, this model complies with biological rules, in particular the 1/3 power law. Two configurations were used here: 1) circular kinematics, with relative phase Φ = 90°, leading to a constant velocity profile matching the kinematic behavior associated with the displacement along a circle and 2) elliptical kinematics, with Φ = 45°, leading to a time varying velocity profile matching the kinematics associated with the displacement along an ellipse of eccentricity 0.9, which corresponds to the ellipse drawn in the most natural way [15].

**Fig 1:**
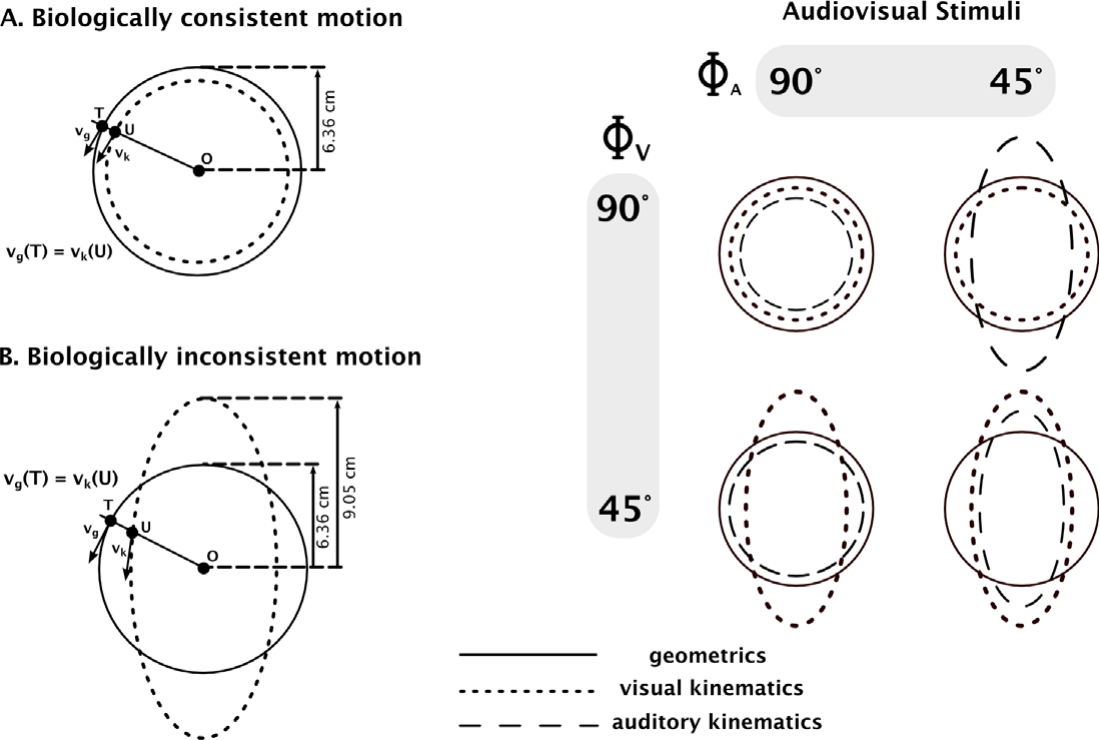
Visual Stimuli. **Left:** Kinematics of visual stimuli (dotted line) that either comply (Panel A) or not (Panel B) with biological rules for a geometric circle (solid line). The velocity of the dot in the biologically consistent motion condition is constant and equals 22.24 cm.s^-1^ and varies between 13.81 cm.s^-1^ and 31.62 cm.s^-1^ for the biologically inconsistent condition. **Right:** Audiovisual stimuli consisting of combinations of visual and auditory motion characterized by their relative phases Φ (indexed V and A for vision and audition respectively).

Visual motions consisted of a white moving dot, with a diameter of 6mm on a black background. The motion was generated according to a method proposed by Viviani, Baud-Bovy, and Redolfi [5]. The dot always followed the same trajectory, a geometric circle with a radius (R) of 6.36 cm corresponding to a circumference of 40 cm, while its velocity varied according to either the circular or the elliptic kinematics as described above (Fig 1, Left). They defined what is referred to as the Visual Kinematics (VK) and were characterized by the relative phase, noted Φ_V_ (indexed V for visual modality). For circular kinematics obtained with Φ_V_ = 90° and A = R corresponding to constant velocity, the kinematics were consistent with biological motion on the circular geometric trajectory traveled by the dot (Fig 1, Left, Panel A). For elliptical kinematics, with Φ_V_ = 45° and A = 6.92 cm corresponding to a time varying velocity, the dot moved faster in the vertical parts of the circle and the motion was therefore biologically inconsistent (Fig 1 Left, Panel B), with the circular trajectory traveled by the dot. Each type of visual motion contained 19 complete cycles of period T = 1.8s and lasted for 34.2s. The four audiovisual stimuli are available in S1–S4 Videos.

Auditory motion was evoked by synthesized friction sounds generated according to a physically-informed model [16,17], which considers that a friction sound is the result of successive micro-impacts produced when a sharp object (exciter) interacts with the asperities of a rough surface (resonator). From a signal point of view, such friction sounds can be simulated by bandpass filtered white noise, in which the central filter frequency is controlled by the velocity profile. A biquad bandpass filter with a constant quality factor equal to 3 was used for this purpose. The relationship between the tangential velocity v_t_ and the central frequency f_c_ of the bandpass filter was defined by: f_c_(t) = α v_t_(t) where α is a constant proportionality coefficient. From a perceptual point of view, the mapping mainly influences the timbre, in particular the mean spectral centroid of the resulting friction sound: the higher the value of *α*, the higher the mean spectral centroid (perceived brightness). The most appropriate mapping (chosen as α = 20) was based on results from a preliminary calibration experiment. For the sake of realism of the synthetic-sound, the contribution of an interacting resonant object was taken into account, and the sounds were synthesised to evoke rubbing on a wooden plate [18,19]. Based on this model, synthesized friction sounds modulated by the velocity profile corresponding either to circular or elliptical kinematics were generated. They defined what is referred to as the Auditory Kinematics (AK) and were characterized by the relative phase, noted Φ_A_ (indexed A for auditory modality). For circular kinematics (Φ_A_ = 90°), the generated sound contained no modulation and evoked a uniform motion, whereas for elliptical kinematics (Φ_A_ = 45°), the generated sound contained acoustic modulations induced by the time-varying velocity profile. These modulations reflect variations of specific acoustical features, such as the spectral centroid that has been shown to adequately evoke perceived motion [20–22].

Hence, 12 different types of audiovisual motion (AVM) were obtained by generating four combinations of constant and time-varying, visual and auditory motions (4 conditions x 3 repetitions, see Fig 1, Right). The AVM had consistent kinematics when both visual and auditory kinematics matched uniform circular kinematics (Φ_V_ = Φ_A_ = 90°). In this case, the AVM corresponded to biological motion. In the three other cases, the AVM had inconsistent kinematics with respect to the geometry as elliptical kinematics were conveyed either in the auditory modality (Φ_V_ = 90°; Φ_A_ = 45°), in the visual modality (Φ_V_ = 45°; Φ_A_ = 90°) or in both modalities (Φ_V_ = Φ_A_ = 45°). These three types of AVM were therefore non biological.

### Task

**Fig 2:**
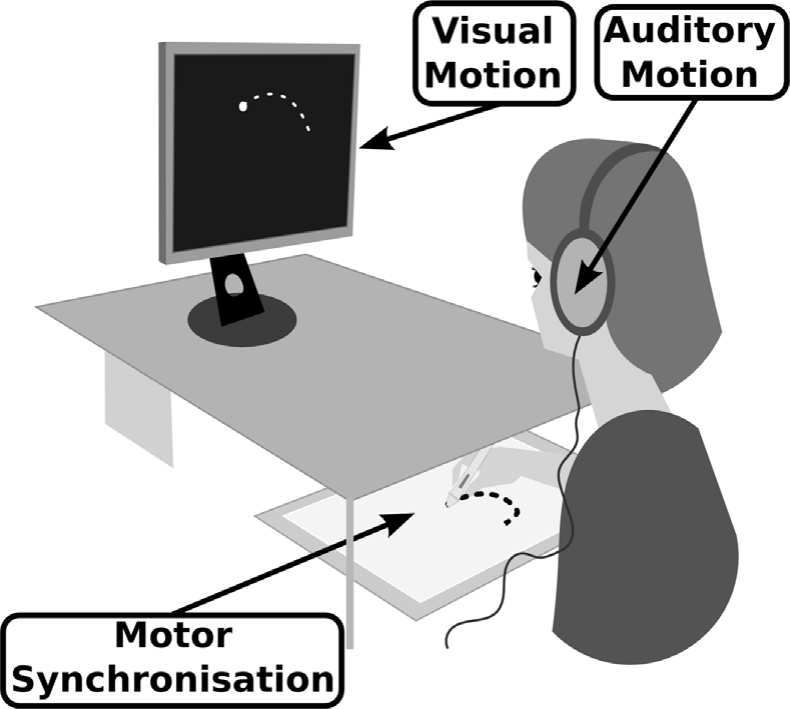
Experimental set-up. The subjects had to synchronize their hand movement with the moving dot on a graphic tablet without seeing their drawing hand.

Subjects were placed in front of a DELL 1907FP screen (1280 x 1024 pixels; 60 Hz refresh rate) that displayed the moving dot while they listened to the auditory stimuli, presented diotically without any spatial cues through a set of headphones (Sennheiser HD650 headphones, the sample rate of the soundcard was 44100 Hz with 16-bit resolution), and were asked to synchronize their hand movement with the visual moving dot on a graphic tablet (Wacom Intuos 5 graphic tablet at a sample rate of 129 Hz and with a spatial precision of 5.10^−3^ mm; see Fig 2). No specific information was given concerning the auditory kinematics or the geometry of the trajectory to be reproduced. In particular, the subjects did not know that the visual trajectory of the moving dot was always circular and that only the kinematics of the moving dot differed. The stimuli were presented according to two different pseudo random series that were balanced across subjects: CC CE EE EE CE EC CC CE EE CC EC CE and EC CC CE EC EC CC EE CE CE EE EE CC, the audiovisual conditions are denoted as follow CC (Φ_V_ = 90°; Φ_A_ = 90°), CE (Φ_V_ = 90°; Φ_A_ = 45°), EC (Φ_V_ = 45°; Φ_A_ = 90°), and EE (Φ_V_ = 45°; Φ_A_ = 45°). A training session was conducted before the experiment to familiarise subjects with the task and with the use of the graphic tablet. During the training session, which contained up to 3 trials of the actual test, the same instructions as in the real test were given. In order to evaluate the visuo-motor coupling without visual feedback on the performed movement, the subjects could not see their hand during the experiment. In order to analyze the influence of the different types of AVM on motor performance, the drawn shapes were fitted to compute their relative phases Φ_drawn_.

### Data Analyses

Data collected on the graphic tablet were filtered using a Savitzky-Golay filter [23] with a 43-point temporal window and third-order interpolation to remove digital noise due to the high sampling rate. Then high-pass Butterworth filtering (0.2 Hz cut-off frequency) was applied to remove the spatial drift observed on hand movements (due to the fact that subjects could not see their hand while drawing). Finally, data were analysed with respect to the relative phase Φ_drawn_ characterising the geometry of the reproduced shape. First, the eccentricity e_drawn_ (i.e. a variable characterising the flatness of the drawn shape) was estimated for the last 10 out of 19 drawn shapes [4,5] and then the relative phase Φ_drawn_ was calculated using the following formula: $\Phi_{\text{drawn}}=2\text{arctan}\sqrt{1-e_{\text{drawn}}{}^{2}}$.

The statistical design contained 2 Visual Kinematics (VK) x 2 Auditory Kinematics (AK). Repeated measures ANOVA was performed with Statistica software to evaluate the effects of each experimental factor on the motor performance described by the relative phase Φ_drawn_. The normality and the homogeneity of the distributions were assessed with a Lilliefors test and a O’Brien test respectively. The distortion between the reproduced shapes and the circular geometry of the visual motion in terms of flatness was performed by means of a one-sample two-tailed t-test between the relative phase Φ_drawn_ and the mean Φ_V_ = 90° in the four audiovisual conditions. The significance level of the p-value was set to 0.05 for all analyses.

## Results

The averaged relative phases by subjects are within S1 Dataset.

**Fig 3:**
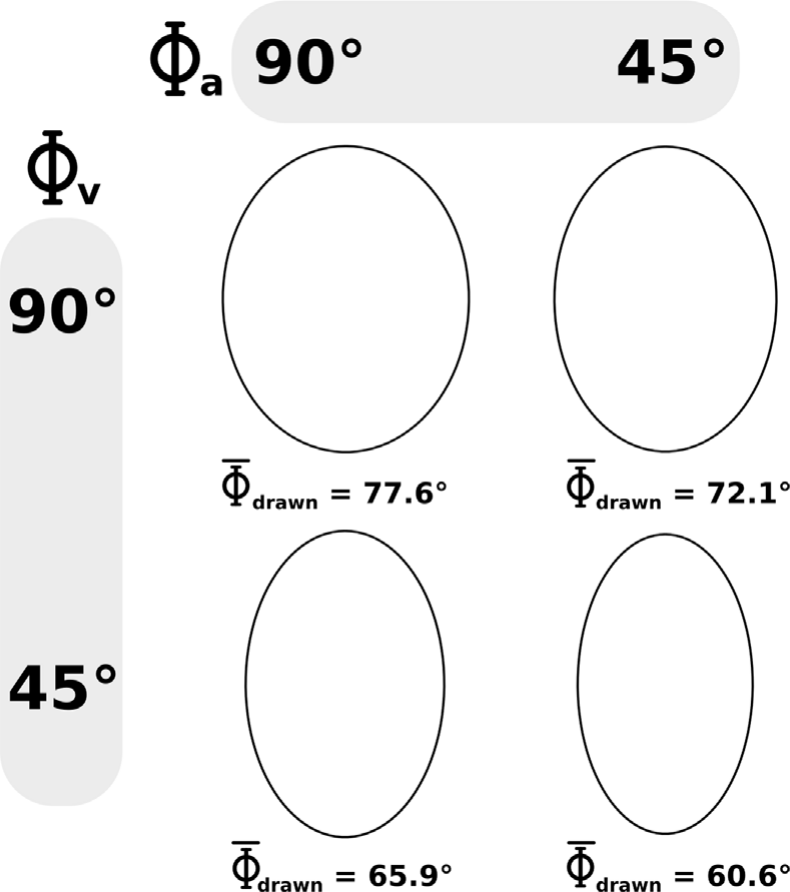
Motor performances. Mean of the motor reproduction in the four audiovisual conditions.

**Fig 4:**
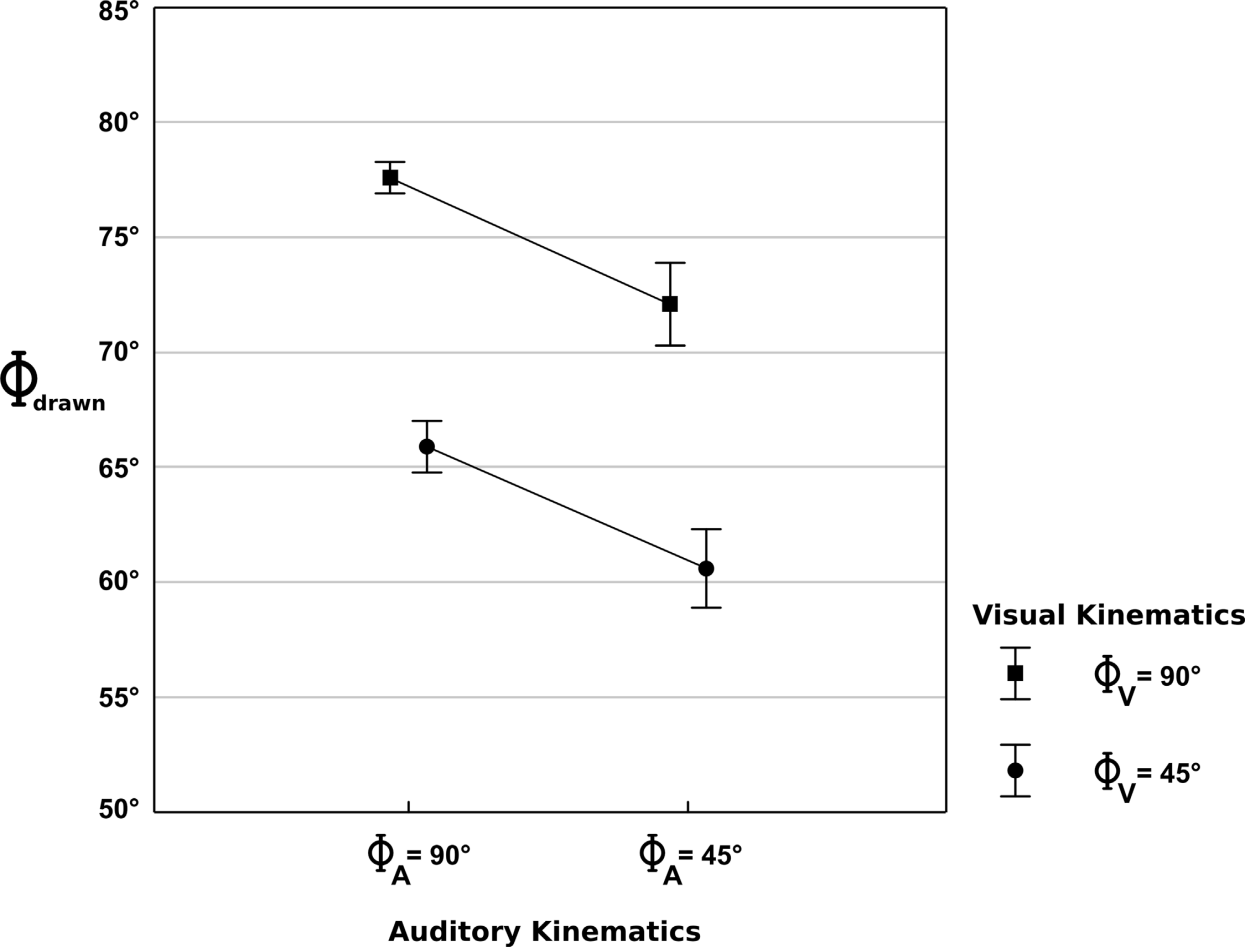
Results. Mean relative phase and 95% confidence intervals of the reproduced motion as a function of visual and auditory kinematics, illustrating the distinct influences of both factors (i.e., no interaction).

The results showed that the geometry of the drawn shapes was noticeably distorted by elliptical kinematics conveyed by both visual and auditory modalities (see Table 1). More precisely a main effect of the visual kinematics (*F*(1,16) = 147.22, *p* < .001) as well as a main effect of the auditory kinematics (*F*(1,16) = 12.83, *p* < .01) on the flatness, characterized by the relative phase of the reproduced shape was revealed, meaning that circles are reproduced flattened (i.e. elliptical) when the visual kinematics (Φ_V_ = 45°) as well as auditory kinematics (Φ_A_ = 45°) are elliptical. However, the interaction between visual and auditory kinematics was not significant (*F*(1,16) = 0.01, *p* = 0.96; Fig 3 and Fig 4). The S1–S4 Videos display the 4 averaged reproduced ellipses along with the corresponding audiovisual stimuli.

**Table 1:** Results of the experiment.

**Φ**_**V**_	**90°**		**45°**	
**Φ**_**A**_	**90°**	**45°**	**90°**	**45°**
**Φ**_**drawn**_	77.6	72.1	65.9	60.6
	[76.1 79.0]	[68.3 75.9]	[63.6 68.3]	[57.03 64.31]
**e**_**drawn**_	0.594	0.685	0.761	0.811
	[0.566 0.622]	[0.625 0.734]	[0.734 0.784]	[0.777 0.839]

Means and 95% Confidence Intervals of the Relative Phases and Eccentricities Characterizing the Drawn Shapes for the Four Conditions.

Figs 3 and 4 show that even in the consistent situation when both auditory and visual kinematics are circular, the reproduced circles were distorted into a more elliptical shape (Φ_drawn_ = 77°). Globally, elliptical visual kinematics (Φ_V_ = 45°) induced stronger distortions than elliptical auditory kinematics (Φ_A_ = 45°). A distortion of 11.7° with respect to the congruent situation was observed when elliptical visual kinematics is combined with circular auditory kinematics (Φ_V_ = 45°, Φ_A_ = 90°), while a distortion of 5.5° is observed when elliptical auditory kinematics is combined with circular visual kinematics (Φ_V_ = 90°, Φ_A_ = 45°). When both visual and auditory kinematics were elliptical (Φ_V_ = 45°, Φ_A_ = 45°), a distortion of 17° was obtained, which corresponds to the sum of the previously observed distortions obtained when either the visual or the auditory kinematics were elliptical. Hence, the distortion induced by elliptical auditory kinematics has not been influenced by visual kinematics (5.3° for Φ_V_ = 45° and 5.5° for Φ_V_ = 90°) and vice versa (11.5° for Φ_A_ = 45° and 11.7° for Φ_A_ = 90°). Finally, the reproduced motion was distorted in all situations (Two-tailed t-tests between Φ_drawn_ and 90°: Φ_V_ = 90°, Φ_A_ = 90°: *t*(16) = -18.09, *p* < .001; Φ_V_ = 90°, Φ_A_ = 45°: *t*(16) = -9.89, *p* < .001; Φ_V_ = 45°, Φ_A_ = 45°: *t*(16) = -21.37, *p* < .001; Φ_V_ = 45°, Φ_A_ = 90°: *t*(16) = -17.06, *p* < .001).

## Discussion

In this study we investigated the influence of both visual and auditory dynamics on the reproduced geometry of visual motion by asking subjects to synchronize drawing movements on a graphic tablet. The results were analyzed in terms of relative phase distortion of the reproduced shape with respect to the visual (circular) trajectory displayed on a screen in front of the subjects.

First, it was found that for all of the audiovisual conditions, the motor reproductions were significantly flatter than the actual circular motions displayed on the screen. This observation is in line with studies on movement coordination showing that when someone draws a repetitive enclosed shape like a circle, he/she naturally tends to draw an elliptical shape which may be considered as an attractor of such a dynamic system [15,24,25].

The results showed that elliptical visual kinematics flattened the reproduced circles regardless of the auditory kinematics. This effect of biologically inconsistent visual kinematics on the reproduced circle underlined the links between motor output and visual motion perception, confirming the results and expectations of Viviani and colleagues [2,3,5]. Our results, issued from a situation without visual feedback from the drawing movement, thereby showed that the motor reproduction of visual biological motion is constrained by co-variations between curvature and kinematic properties in a feedforward manner, which could not have been fully demonstrated in the visual feedback condition investigated by Viviani et al. [2].

However, it must be noted that we cannot conclude whether both visual perception and motor processes are affected by incongruent auditory motion as our experiments were performed in a multisensory and motor context. Nevertheless, we recently showed [5] that distortions induced by elliptical visual kinematics (Φ_V_ = 45°) are larger for the visuo-motor coupling (10.17%) than the perceptual distortions observed by Viviani and colleagues in purely visual (1.01% in [3]) or kinaesthetic presentations (<1% in [5]). As we here observed that the motor reproduction was less distorted by auditory elliptical kinematics (Φ_A_ = 45°) than elliptical visual kinematics (Φ_V_ = 45°), we may expect that if distorted, the perception of a visual circular motion (Φ_V_ = 90°) would be less affected by elliptical auditory kinematics (Φ_A_ = 45°) than by elliptical visual kinematics (Φ_V_ = 45°).

The key point of this study is that continuous friction sounds also substantially interfered with visual motion cues and clearly modified the geometric properties of the motor reproduction by flattening the reproduced circles regardless of the visual kinematics in the biologically inconsistent condition. In line with our hypothesis, this effect of sound on the reproduced geometry strongly attests to the central role of dynamics *per se*, on the visuo-motor coupling. While the influence of visual kinematics involves both geometric and kinematic cues, the influence of auditory dynamics evoked by timbre variations of friction sounds allows for the investigation of the role of kinematic cues alone and reveal that the emergence of the geometry and the associated motor output is clearly driven by motion dynamics. Note that this would not have been possible to investigate the role of auditory dynamics with rhythmic discrete sounds, e.g. corresponding to velocity minima or maxima, as such stimuli convey only the timing of the movement and not its continuous velocity variations. Hence, while the studies made by Viviani et al. [2,3,5] supporting perceptual-motor interactions were performed in a purely unimodal context, in the present study we showed that such interactions also can occur in a multisensory context. In line with this, Varlet et al. [26] investigated sensorimotor coordination between audio-visual motion and a motor synchronization. They reported that continuous sounds affect motor synchronization. Although their study did not focus on the geometry but on temporal aspects of motor synchronization with audiovisual stimuli on a horizontal axis, their results highlighted the influence of motor attractors and biomechanical constraints in the sensori-motor coupling with audiovisual motions.

The absence of interaction between visual and auditory kinematics in our study suggests that the visual and auditory influences were combined in a perfectly linear way. While both the visual and auditory motions were inconsistent, i.e. elliptical (Φ_V_ = 45°, Φ_A_ = 45°), the distortion of the reproduced shape equaled the sum of the individual distortions obtained when either the visual motion was biologically inconsistent (Φ_V_ = 45°, Φ_A_ = 90°) or the auditory motion was inconsistent (Φ_V_ = 90°, Φ_A_ = 45°). Although the literature on multisensory integration most often claims that the effects of several sensory channels add up non-linearly and that synchronous sensory inputs amplify or inhibit perceptual and behavioral effects (e.g., [27]), some behavioral studies did not support this view and revealed additive effects [28–31]. Here, we provide a clear example of linear integration between visual and auditory motion-related cues. More than the additivity of the effects observed here, it is noticeable that the distortion induced by biologically inconsistent kinematics in the visual domain is stronger (about 12°) than the distortion induced by inconsistent auditory kinematics (about 5°). This suggests that the effect of dynamics per se is about half the combined effect of spatial and dynamic cues.

Taken together, these results could be interpreted in line with the Theory of Event Coding [32] stressing the existence of unified percepts that combine both sounds produced by objects and modulated by actions with their perceptual properties. Thoret et al. [13] suggested the existence of such a unified percept of biological movements from a purely auditory point of view, i.e. linking the properties of drawing movements to their evocation through timbre variations of friction sounds. Studies by Danna et al. [33] confirmed such binding between friction sounds and drawing movements by showing that the quality of handwriting can be accurately judged through friction sounds evoking the movement velocity. Such unified percepts that link sounds and actions have also been shown for footstep sounds [34]. Our results are clearly in line with the existence of this kind of unified percept in a multisensory context involving audio-visuo-motor coupling. In particular, the motor production appears to be driven by the linear integration of both geometric and dynamic cues in the visual modality and solely by dynamic cues evoked through timbre variations in the auditory modality.

Finally, these findings may help in designing new devices using sounds to enhance or substitute visual and, more generally, sensory information. In particular, training activities involving continuous auditory stimuli when treating diseases that affect motor function, such as dysgraphia [35] and Parkinson’s disease [36,37], or when guiding movements in sport [38], are believed to constitute powerful alternatives to existing methods. In this context, we suggest that conveying dynamic information through specific sound properties can substantially modify motor performance.

## Conclusions

The experiment presented in this study provides an evidence of the central role of dynamics in the reproduction of the geometry of a visual motion in an audiovisual context. We showed that both biologically inconsistent visual motion and inconsistent auditory motion flatten the reproduced geometry of circular visual motions. Interestingly, the combined effects of visual and auditory inputs observed here were added up linearly. Nevertheless, in order to better understand these effects and how the two modalities are combined to plan motor actions, it might be interesting to manipulate the instructions given to the subjects. This could be done by asking subjects either to focus on the sound rather than the visual motion, or to focus with the same attention on the visual and auditory information. The experimental data could be analyzed in terms of sensorimotor coordination such as in the study of Varlet et al. [26]. The relative phase considered in our study as a geometrical descriptor characterizing the eccentricity of the ellipse could then be used to reveal the temporal coordination of the movements in terms of negative versus non-negative lag and stability, which might also be affected by incoherent audiovisual motions. Moreover, purely perceptual experiments investigating whether incongruent audiovisual motions affect the visual perception of the geometry would enable to reveal information on the separate role of perceptual and motor processes in such a task.

In a more general perspective, it might be of interest to evaluate whether the effects observed here are specific to biological motions or whether the motor reproduction of any kind of audiovisual motion might be affected by incongruent visual and auditory kinematics, for instance by considering physical motions such as those constrained by Newton’s laws [39].

## Supporting Information

S1 Video Audiovisual stimulus (Φ_V_ = 90°; Φ_A_ = 90°) and the corresponding averaged reproduced movement. The visual sample rate of the original visual stimulus has been downsampled to 30 Hz and the audio encoded in the AAC format due to technical capabilities.
(MP4)

S2 Video Audiovisual stimulus (Φ_V_ = 90°; Φ_A_ = 45°) and the corresponding averaged reproduced movement. The visual sample rate of the original visual stimulus has been downsampled to 30 Hz and the audio encoded in the AAC format due to technical capabilities.
(MP4)

S3 Video Audiovisual stimulus (Φ_V_ = 45°; Φ_A_ = 90°) and the corresponding averaged reproduced movement. The visual sample rate of the original visual stimulus has been downsampled to 30 Hz and the audio encoded in the AAC format due to technical capabilities.
(MP4)

S4 Video Audiovisual stimulus (Φ_V_ = 45°; Φ_A_ = 45°) and the corresponding averaged reproduced movement. The visual sample rate of the original visual stimulus has been downsampled to 30 Hz and the audio encoded in the AAC format due to technical capabilities.
(MP4)

S1 Dataset Averaged relative phases by subjects. (TXT)
